# Single-cell RNA-seq uncovers lineage-specific regulatory alterations of fibroblasts and endothelial cells in ligamentum flavum hypertrophy

**DOI:** 10.3389/fimmu.2025.1569296

**Published:** 2025-05-15

**Authors:** Yongxin Chen, Jue Zhang, Xincheng Feng, Qinghong Ma, Chao Sun

**Affiliations:** Department of Spine Surgery, The Affiliated Jiangning Hospital of Nanjing Medical University, Nanjing, Jiangsu, China

**Keywords:** lumbar spinal stenosis, hypertrophy, scRNA-seq, fibroblast, endothelial cell, ligamentum flavum

## Abstract

**Background:**

Lumbar spinal stenosis (LSS) represents a major global healthcare burden resulting in back pain and disorders of the limbs among the elderly population. The hypertrophy of ligamentum flavum (HLF), marked by fibrosis and inflammation, significantly contributes to LSS. Fibroblasts and endothelial cells are two important cells in the pathological process of ligamentum flavum (LF) fibrosis and inflammation. These two cells exhibit heterogeneity in various fibrotic diseases, yet their heterogeneity in LF fibrosis remains poorly defined.

**Methods:**

Using single-cell RNA-seq, we examined the alterations of fibroblasts, endothelial cells, and key genes in the hypertrophic LF, aiming to establish a comprehensive single-cell atlas of LF to identify high-priority targets for pharmaceutical treatment of LSS.

**Results:**

Here, we find there are five distinct subpopulations of LF fibroblasts: secretory-papillary, secretory-reticular, mesenchymal, pro-inflammatory, and unknown. Importantly, in HLF, the proportion of mesenchymal fibroblast subpopulations increases significantly compared to normal LF (NLF), reflecting their close association with the pathogenesis of HLF. Furthermore, critical target genes that might be involved in HLF and fibrosis, such as MGP, ASPN, OGN, LUM, and CTSK, are identified. In addition, we also investigate the heterogeneity of endothelial cells and highlight the critical role of AECs subpopulation in LF fibrosis.

**Conclusion:**

This study will contribute to our understanding of the pathogenesis of HLF and offer possible targets for the treatment of fibrotic diseases.

## 1 Introduction

Lumbar spinal stenosis (LSS) ranks as a frequent ailment among the aging population (1, 2). The disease usually causes pain in the lower back that radiates bilaterally up to the buttocks and lower limbs, numbness, lameness, and limited mobility, resulting in a heavy socio-economic burden worldwide (2–4). This disorder encompasses a multitude of pathological mechanisms, including degeneration of the intervertebral disc, osteophytes, and hypertrophy of ligamentum flavum (HLF) (5, 6). Among them, the HLF has been identified as a major contributor to the etiology of LSS (7).

Previous studies have demonstrated that HLF mainly displays fibrotic alterations, which are typically characterized by elevated collagen fibers and diminished elastic fibers (6–8). The defining features of fibrotic diseases include fibroblast growth and the overaccumulation of local inflammation and extracellular matrix (ECM) (9–13). LF fibrosis progresses as a result of chronic inflammatory response. However, there are currently no effective medications to slow or stop the ongoing worsening of LF fibrosis and inflammation (14), and the molecular pathways underlying the disease are still mostly unknown.

Previously, it was found that some cells, including endothelial cells and fibroblasts, have been recognized as contributors to fibrosis (7–10, 13). Fibroblasts and endothelial cells are two primary types of cells responsible for ECM buildup and local inflammation (7–10, 13), but their heterogeneity in LF fibrosis remains poorly understood. Single-cell RNA sequencing (scRNA-seq) has become extensively utilized in recent years to investigate biological processes and discover novel cell subtypes in various organs and tissues such as the uterus, kidney, liver, and skin (15–17). Interestingly, there is growing evidence that cell heterogeneity is present in some fibrotic diseases and plays a critical role in the pathological process of fibrosis (17–19).

In the current work, we performed scRNA-seq on NLF and HLF. The current study is refined and developed through a fresh perspective of interpretation. It also reveals for the first time the heterogeneity of ECs during fibrosis of the LF. Our findings indicated that there were five subpopulations of LF fibroblasts. HLF contained a significantly higher proportion of fibroblasts that expressed mesenchymal cell markers than the NLF. Subsequent research indicated that the overexpression of collagens in HLF might be caused by this subpopulation of fibroblasts and may be associated with the abnormal expression of MGP, ASPN, OGN, LUM, and CTSK. Additionally, we also explored endothelial cell (EC) heterogeneity to more fully understand LF fibrosis. The results indicated the proportions of arterial endothelial cells (AECs) subpopulation were significantly increased in HLF. Furthermore, we discovered that the AECs subpopulation was enriched for LF fibrosis-related processes. These discoveries will deepen our understanding of HLF and offer possible targets for treatments for fibrosis.

## 2 Methods

### 2.1 Participants

We enrolled one patient with LSS and one patient with LDH for this study. **Supplementary Table S1** provides details of the patients. Each participant provided written consent after being informed. Approval for this study was granted by the institutional review boards of Nanjing Medical University (Approval No. 2022-03-01-H04).

### 2.2 LF specimen collection

All LF specimens were obtained from the L4/5 segment during spinal decompression surgery. The surgery was performed by three experienced spine surgeons. After the fresh tissue was removed from the human lumbar spine, the surrounding fat and other impurities were removed and rinsed thoroughly with saline, placed in ice-cold PBS, and transferred directly to the dissociation laboratory. On T2-weighted MRI images at the L4/5 facet joints, the LF thickness over 4 mm was classified as HLF, and the LF thickness less than 4 mm was classified as NLF. Patients with spondylolisthesis, spinal tuberculosis, ankylosing spondylitis, spinal tumors, or spinal infections were excluded.

### 2.3 Dissociating tissues and preparing single-cell suspensions

The LF specimens were sliced into pieces measuring 0.5 mm² and washed repeatedly with 1× PBS to remove surface impurities. The treated LF tissues were added to the dissociation solution, and the process was executed at 37°C in a water-bath shaker for a duration of 20 minutes. After filtering the cell suspension through a stacked cell strainer, it was centrifuged at 300 g for 5 minutes at 4°C. Then, the cell precipitate was collected and resuspended with PBS solution. Lysis buffer was added on ice to remove red blood cells, followed by centrifugation at 300 g for 5 minutes, and the cell pellet was collected. Dead cells were removed using the Miltenyi^®^ Dead Cell Removal Kit (MACS 130-090-101). Following this, the suspension was resuspended in 0.04% BSA and centrifuged at 300 g for 3 minutes at 4°C, with the procedure repeated twice. After obtaining the candidate cells, 50 μl of 0.04% BSA was used to prepare a cell suspension. Taipan blue staining was used to detect cell activity, which needed to be greater than 85%. The cell concentration, measured by an Automated Cell Counter, was between 700 and 1200 cells per microliter.

### 2.4 Chromium 10x genomics library and sequencing

According to the manufacturer’s guidelines for the Human Single-Cell Kit, single-cell suspensions were loaded onto 10x Chromium to capture individual cells. The steps for cDNA amplification and library construction that followed adhered to the designed protocol. Sequencing of the libraries was performed at Nanjing Medical Corporation using the paired-end mode on a HiSeq Xten (Illumina, USA). Technical assistance is supplied by Shanghai Baimu Biotechnology Co.

### 2.5 Bioinformatics analysis

FASTQ format is used to store sequenced reads from single-cell sequencing data. Raw FASTQ sequencing data were processed using CellRanger (V6.1.1), a software officially provided by 10x Genomics for 10x single-cell transcriptome data analysis. Normalize gene expression matrices using Seurat’s “LogNormalize” method. Use Seurat (V4.1.0) to extract cell-to-cell differential genes and eliminate sources of variation (e.g., technical noise, cell cycle, etc.). In Seurat, PCA principal component analysis was performed to select the 23 principal components with the highest contribution (cumulative contribution of variance > 85%), and cluster analysis was performed using the graph-based algorithm. The UMAP dimensionality reduction algorithm was used to show the cell distribution in a 2D space. Cells in the same cluster are represented by the same color. The Seurat parameter resolution is generally defaulted to 0.6. Marker genes were identified using Seurat’s Wilcoxon rank sum test (∣log_2_FC∣ > 0.25, P. adj < 0.05). Cell type annotation was performed using the celliD (V1.8.1) package. The analysis results were visualized using the official 10X Genomics LoupeBrowser software.

### 2.6 Gene sets enrichment analysis

GO functional enrichment and KEGG pathway enrichment of differentially expressed gene sets between groups were performed using ClusterProfiler (V4.2.2) software based on the principle of hypergeometric distribution. Significant enrichment was defined as P. adj < 0.05. Gene Set Enrichment Analysis (GSEA) analysis was performed using GSEA desktop software. The normalized enrichment score (NES) takes into account both the number of gene sets and the number of genes. Pathways with |NES| > 1, p-value < 0.05, and q-value < 0.25 were defined as significantly enriched.

### 2.7 Pseudotime analysis and RNA velocity analysis

Use Monocle 2 to analyze patterns of changes in gene expression to reconstruct cellular developmental trajectories to reveal evolutionary pathways and critical turning points in different states of the cell. Using the velocyto R package to distinguish between unspliced mRNA and spliced mRNA, it is possible to understand changes in mRNA velocity and assess the level of gene transcription and expression. The combination of mRNA velocities can then be used to estimate the future state of individual cells and infer cellular developmental trajectories. Visualization using Seurat (V 4.1.0).

### 2.8 Cell–cell communication analysis

CellPhoneDB 2.0 is a tool to study intercellular communication based on cell matrix data, which takes into account the structural composition of ligands and receptors, allows for a comprehensive and systematic analysis of intercellular communication molecules, and the study of intercommunication and communication networks between different cell types. We used CellPhoneDB 2.0 to analyze the receptor and ligand expression information of cells to reveal the interactions between different cell types. CellPhoneDB 2.0 results were visualized using the scPRIT package, and heatmaps, dot plots, and chord plots were drawn.

### 2.9 Identification of key genes and PPI network

The PPI network was constructed using the Search Tool for Retrieval of Interacting Genes (STRING) database. The visualization of PPI networks was performed with Cytoscape (V3.10.3). Hub genes in the PPI network were detected by the maximum clique centrality (MCC) algorithm, which is a form of the cytoHubba plug-in. PyMOL (V3.1.3) visualized LUM and COL1A2 binding patterns predicted by the HDOCK server.

### 2.10 Transcription factor regulatory network analysis

The NetworkAnalyst tool (https://www.networkanalyst.ca/) was used to analyze interactions between the transcription factors (TFs) and hub genes, which were then visualized with Cytoscape (V3.10.3). The latest pySCENIC pipeline was used for single-cell regulatory network inference and clustering (SCENIC) analysis, uncovering TF expression and regulation across various cell groups.

### 2.11 Histological analysis

After surgery, human LF specimens were immediately fixed in 4% paraformaldehyde. Following a three-week decalcification process, the specimens were paraffin-embedded, then sliced into 4-μm sections. As directed by the manufacturer, the sections were deparaffinized and hydrated before being stained using H&E staining kits (Servicebio, China), EVG staining kits (Servicebio, China), and Masson trichrome staining kits (Servicebio, China).

### 2.12 Immunohistochemistry staining

LF tissues were preserved in 4% paraformaldehyde, embedded in paraffin, and sliced into 4 μm thick sections. Antigen retrieval was carried out using an EDTA buffer, followed by a 10-minute treatment with a 3% hydrogen peroxide solution at room temperature, and subsequently blocked with 5% BSA. Afterward, sections were incubated overnight at 4°C with antibodies for α-SMA, COL1A2, COL3A1, MGP, ASPN, OGN, LUM, and CTSK (Abcam). Following PBS washing, the LF sections underwent incubation with a secondary antibody. The color was subsequently developed using a DAB solution. The sections were counterstained with hematoxylin and sealed for subsequent observation.

### 2.13 Immunofluorescence staining

The LF cells were treated with 4% paraformaldehyde at room temperature for 10 minutes, then permeabilized using 0.1% Triton X-100 for 15 minutes, and subsequently blocked with 5% goat serum for 1 hour. Afterward, the cells were left to incubate with antibodies targeting FIBIN and α-SMA (Abcam) overnight at 4˚C. Ultimately, the LF cells were kept in the dark for one hour with FITC- or Cy3-linked anti-rabbit IgG secondary antibodies from Abcam. DAPI (Beyotime, China) was applied to stain the nuclei for a duration of 10 minutes. Images were taken and examined using a fluorescence microscope (Olympus, China).

### 2.14 RT-PCR

TRIzol reagent (Invitrogen, USA) was used to extract total RNAs from human LF specimens. Then, the conversion of total RNAs to cDNA was performed using the Takara kit. Gene expression levels were assessed with the Real-Time System for Thermal Cycler Dice (Takara). Relative levels of mRNA expression were calculated using the 2^-DDCT^ method with GAPDH as an internal control. Listed in **Supplementary Table S2** are the primers and sequences for the specific designs.

### 2.15 Statistical analysis

GraphPad Prism software (San Diego, CA, USA) was used to conduct statistical analysis and create graphs. A Student’s t-test was utilized to analyze quantitative data and compare values between two groups. At P < 0.05, the difference was statistically significant.

## 3 Results

### 3.1 ScRNA-seq atlas of LF cell populations

**Figure 1:**
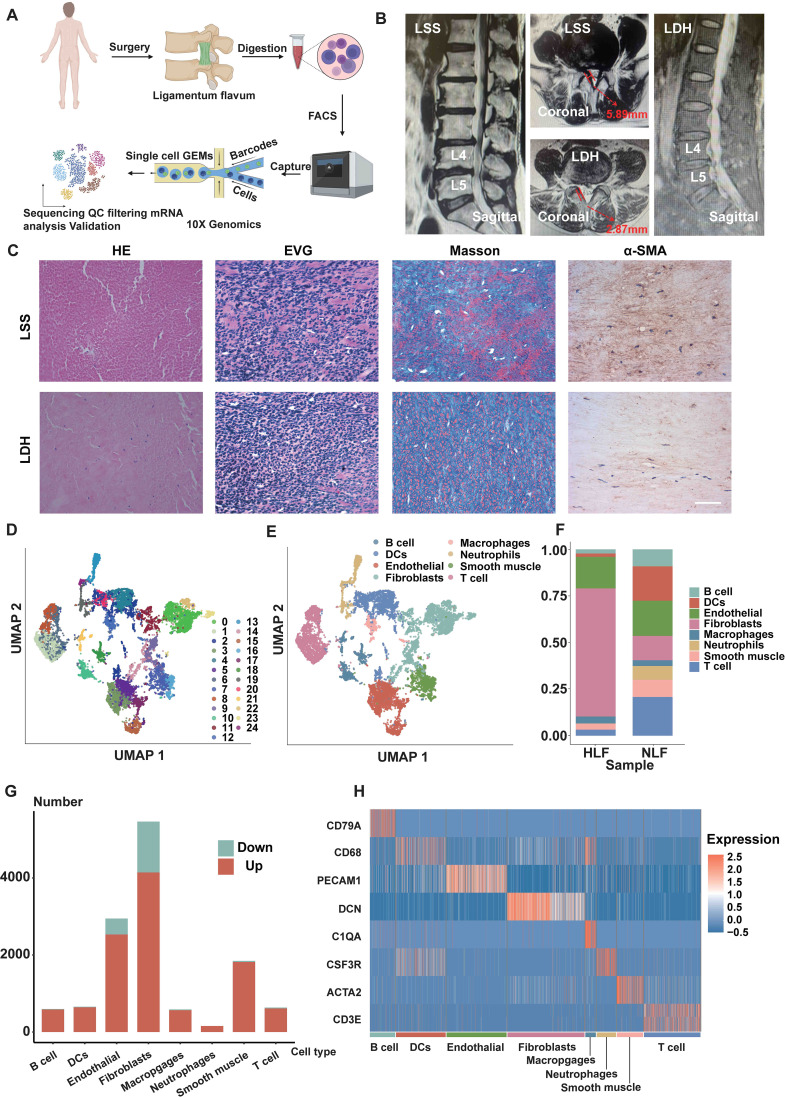
Single-cell atlas of NLF and HLF specimens. **(A)** Overview of the workflow of scRNA-seq in human LF tissues. **(B)** Measurement of human LF thickness at the level of the facet joints marked by the red arrows on MRI. **(C)** Images of LF specimens stained with H&E, EVG, and Masson and representative images of IHC staining of a-SMA. Scale bar, 100 μm. **(D)** An unbiased analysis of 11190 cells identifies 25 cellular clusters, each represented by a different color, with their general identities displayed on the right. **(E)** The 25 cellular clusters are divided into 8 cell lineages, with each cell lineage marked by different colors. The overall identity of each cell cluster is indicated above. **(F)** Comparison of cell lineage proportions between NLF and HLF. **(G)** Amount of differentially expressed genes in each cell type with more than 30 cells in NLF and HLF (adjusted P-value of <0.05). Red bars represent genes that are upregulated, while gray bars represent genes that are downregulated in LF. **(H)** Distribution heatmap of expression levels for genes particular to selected clusters. LF, ligamentum flavum; NLF, normal ligamentum flavum; HLF, hypertrophy of ligamentum flavum.

To analyze the cell heterogeneity and investigate regulatory alterations in LF fibrosis, we obtained fresh LF from an LSS patient as well as a lumbar disc herniation (LDH) patient and performed single-cell RNA sequencing (**Figure 1A**). Previously, it was well demonstrated that patients with LSS had significantly thicker LF, and fibrosis was the primary pathological characteristic of HLF (7, 8, 20). As such, the MRI findings exhibited the LF thickness in patient with LDH was 2.87 mm, whereas the LF reached 5.89 mm thick in patient with LSS. The LF was significantly thicker in the LSS patient (**Figure 1B**). Morphologically, it was found that elastic fibers were abundant and neatly aligned in the NLF tissue from the LDH patient according to H&E and EVG staining. However, the HLF from the LSS patient revealed a noticeable decrease of elastic fibers (**Figure 1C**). In Masson staining, collagen fibers appeared blue, whereas elastic fibers were pink. A significant percentage of the LDH specimen was stained pink. Nonetheless, the majority of the LSS specimen had blue staining, also indicating fibrotic alterations (**Figure 1C**). Furthermore, immunohistochemical (IHC) analysis revealed that the LSS specimen exhibited a higher accumulation of a-SMA protein compared to the LDH specimen (**Figure 1C**).

**Figure 2:**
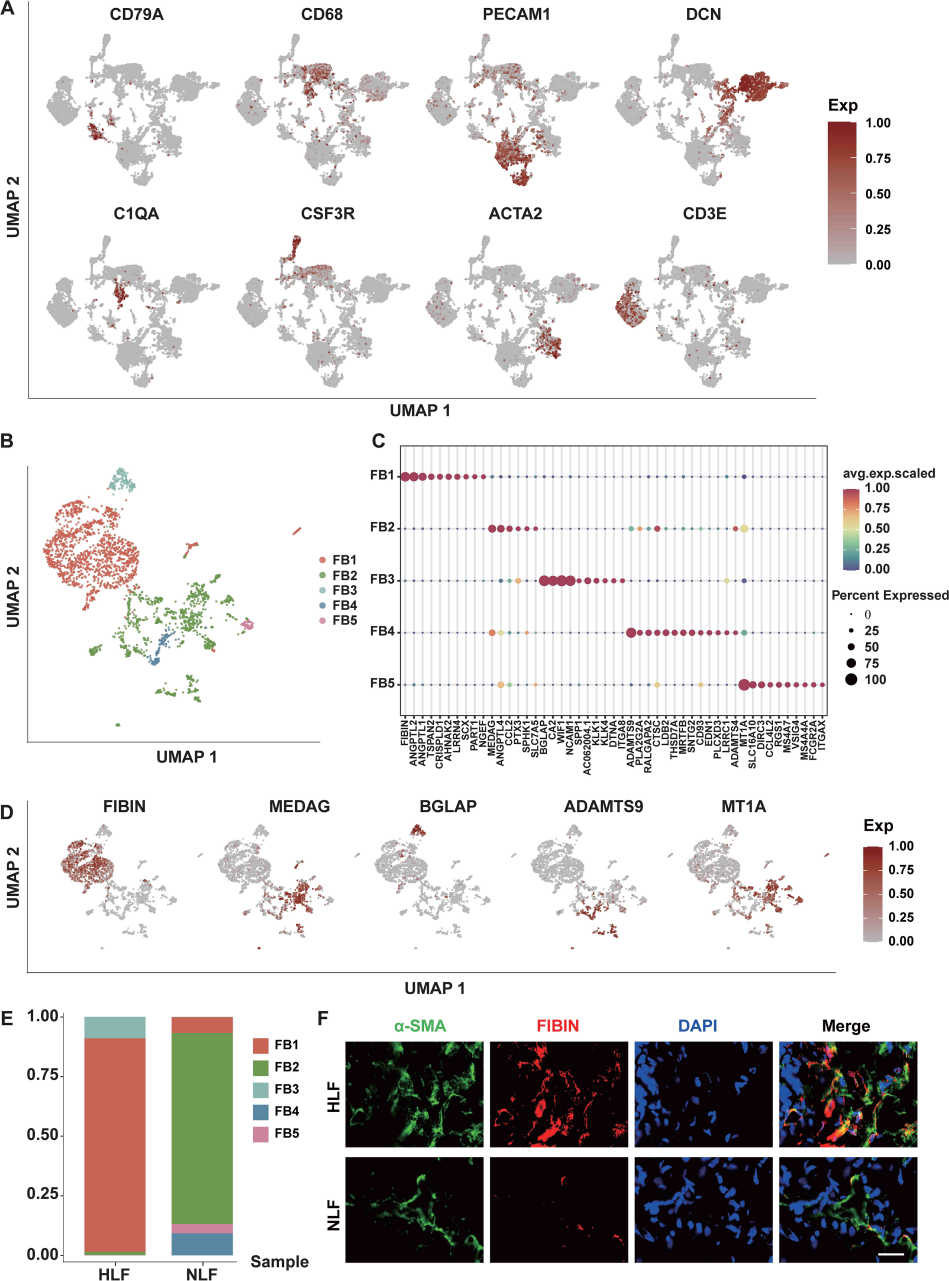
LF fibroblasts exhibit heterogeneity. **(A)** UMAP plots illustrate the distribution of lineage-specific gene expression, with cells color-coded by their expression levels. **(B)** Five distinct fibroblast subpopulations were identified in LF specimens. **(C)** Graphs showing differentially expressed genes. For each subpopulation, the leading 10 genes and their expression levels among all fibroblasts are displayed. Displayed on the right are the genes chosen for each cluster, with color coding. **(D)** Plots illustrating the expression distribution of subpopulation markers. Expression levels for each cell are color-coded and overlaid onto the UMAP plot. **(E)** Analyzing the percentage of five fibroblast subpopulations between NLF and HLF. **(F)** Double-immunofluorescence staining exhibits the localization of a-SMA (green) and FINIB (red) in LF tissues from the NLF and HLF. Scale bar, 100 μm. NLF, normal ligamentum flavum; HLF, hypertrophy of ligamentum flavum.

After stringent quality control, scRNA-seq of two specimens yielded 11,190 total cells (HLF: 2,144; NLF: 9,046) for further analysis. The UMAP clustering technique uncovered 25 distinct cell clusters (**Figure 1D**). We identified fibroblast clusters and endothelial cell (EC) clusters, which together constituted over 90 percent of sequenced cells. Using known marker genes specific to each lineage, we divided these clusters into 8 distinct cell lineages (**Figure 1E**). DCN was employed to identify the fibroblast lineage, while PECAM1 was utilized to identify the endothelial lineage (**Figures 1H**, **2A**).

Furthermore, we compared the percentage differences among all cell lineages in HLF and NLF. Significant variations were observed in the relative cell number ratios between the HLF and NLF cell lineages (**Figure 1F**). Notably, HLF had a higher ratio of fibroblasts and a lower ratio of endothelial cells than NLF (**Figure 1F**). ECs were diminished in HLF potentially due to the excessive proliferation of fibroblasts. Alternatively, this may be due to the fact that during HLF, the ECs are transferred to fibroblast cells, which is also known as endothelial-to-mesenchymal transition (EndMT) (21, 22). Subsequently, we explored the quantity of differentially expressed genes (DEGs) between HLF and NLF (**Figures 1G, H**). Likewise, the results suggested that fibroblasts showed the greatest changes, indicating significant alterations in fibroblasts during LF fibrosis.

### 3.2 LF fibroblasts subcluster into distinct cell populations and mesenchymal fibroblasts are increased in HLF

Due to the substantial alterations of fibroblasts (**Figures 1F, G**, **2A**) during the fibrotic progression in HLF and their critical role in fibrotic pathogenesis (23, 24), we next performed a cluster analysis of all HLF and NLF fibroblasts to further reveal their heterogeneity. Hierarchical cluster analysis indicated that fibroblasts can be further categorized into five subpopulations: mesenchymal, pro-inflammatory, secretory-papillary, secretory-reticular, and unknown (**Figure 2B**). Afterwards, we derived the 10 most significantly expressed genes in each subpopulation by analyzing the average expression levels of each gene and their expression percentages within the cells of each subpopulation (**Figure 2C**). Consequently, the mesenchymal fibroblasts (FB1) subpopulation was identified by FIBIN, the pro-inflammatory fibroblasts (FB2) by MEDAG, the secretory-papillary fibroblasts (FB3) by BGLAP, and the secretory-reticular fibroblasts (FB4) by ADAMTS9 (**Figure 2D**). However, the FB4 subpopulation was not detected in the HLF specimen. The number of cells in unknown fibroblasts (FB5) subpopulation was minimal. Therefore, we excluded it from subsequent subpopulation analyses.

Furthermore, a comparison of differences in the proportions of fibroblast subpopulations indicated a reduction in the proportions of FB2, FB4, and FB5 subpopulations in HLF, while the FB1 and FB3 subpopulations exhibited an increase (**Figure 2E**). The proportion of the FB1 subpopulation amounted to 89.55%. There was a significant increase in the FB1 subpopulation in HLF, which suggests FB1 might significantly influence the progression of HLF. This result was also confirmed by immunofluorescence (IF) staining (**Figure 2F**), which showed that the percentage of FIBIN cells, also known as FB1 cells, was greater in HLF than that in NLF (**Figure 2F**). As is known, α-SMA protein is considered a critical marker for the severity of LF fibrosis (8). IF analysis also indicated a significantly higher presence of the FB1 subpopulation with fibrosis features (FIBIN+α-SMA)^+^ in HLF compared to those from the NLF (**Figure 2F**). According to this finding, it can be concluded that FB1 subpopulations are pivotal cells responsible for LF fibrosis.

### 3.3 Characteristics of mesenchymal fibroblasts in HLF

**Figure 3:**
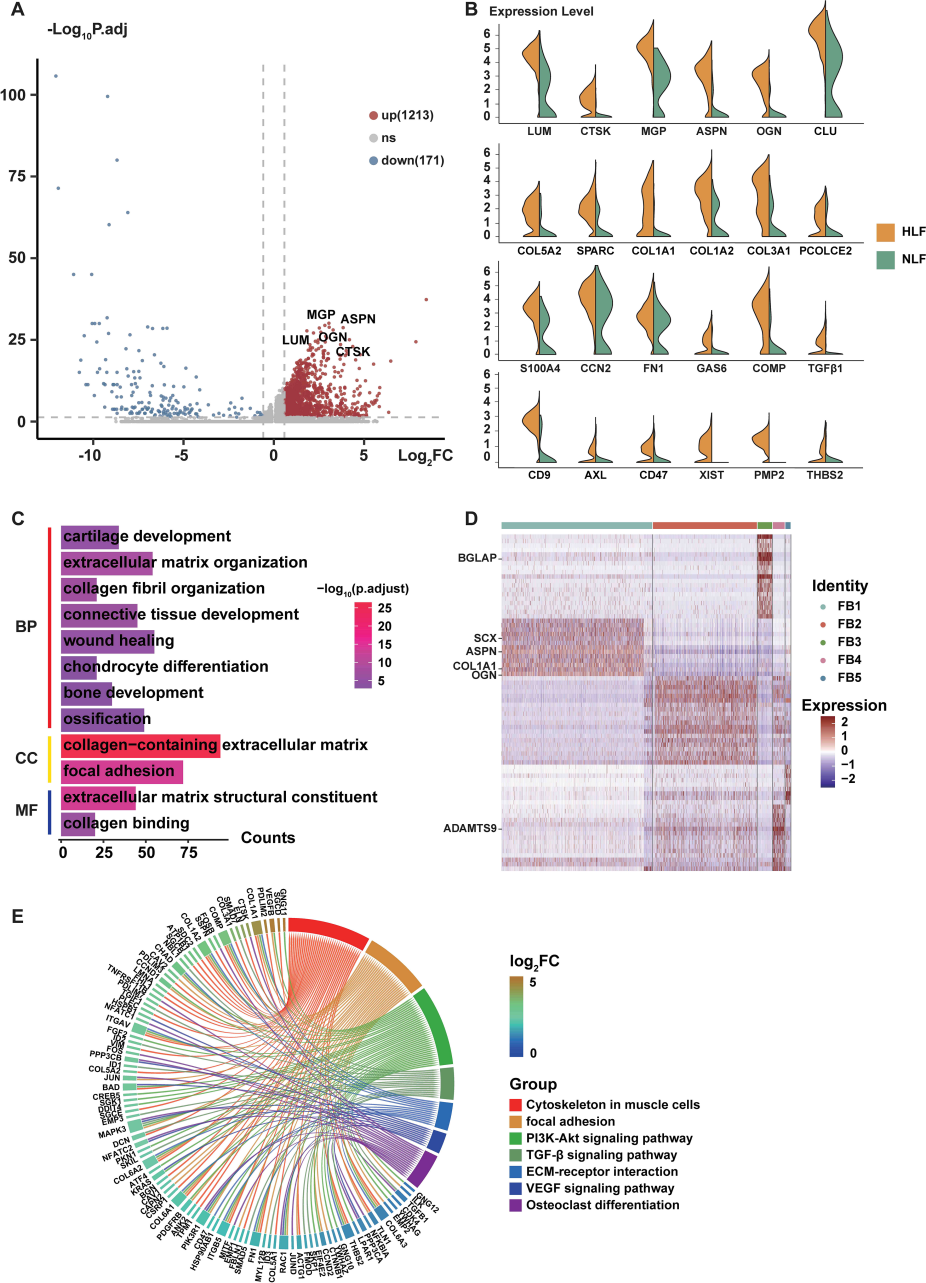
Features of FB1 fibroblasts involved in LF fibrosis. **(A)** The volcano plot for FB1 fibroblasts displays 1213 upregulated genes and 171 downregulated genes. **(B)** Representative genes with differential expression between NLF FB1 and HLF FB1 are shown in violin plots. **(C)** GO enrichment analysis of genes with increased expression in FB1 fibroblasts from NLF and HLF. Significance is determined by an adjusted P-value that is less than 0.05. **(D)** Heatmap displaying differentially expressed genes in HLF fibroblast subpopulations. Gene expression levels are indicated on the right. **(E)** The KEGG enrichment analysis focused on elevated genes in FB1 fibroblasts between NLF and HLF. An adjusted P-value below 0.05 was used as the significance threshold. BP, Biological Process, CC, Cellular Component, MF, Molecular Function; NLF, normal ligamentum flavum; HLF, hypertrophy of ligamentum flavum.

The scRNA-seq data showed a notable rise in the proportion of FB1 in HLF relative to NLF. Therefore, our subsequent studies concentrated on the FB1 and explored the number of DEGs in FB1. The results revealed significant expression alterations of 1,384 genes (up: 1213; down: 171) in the HLF FB1 (**Figure 3A**). Violin plots emphasize the dysregulation of various genes associated with chondrogenesis and ossification (CLU, COMP, PCOLCE2, and SPARC) as well as ECM-related genes (COL1A1, COL1A2, and COL3A1) in the HLF FB1 subpopulations (**Figure 3B**). Additionally, we also found that the levels of some membrane proteins (CD47, AXL, FZD8, and CD9) and myofibroblast markers (FN1, CCN2, and S100A4) were markedly elevated in the HLF FB1 subpopulation as compared to NLF FB1 (**Figure 3B**). GO analysis showed HLF FB1 exhibited enrichment in processes associated with extracellular matrix organization, cartilage development, ossification, chondrocyte differentiation, collagen fibril organization, bone development, wound healing, connective tissue development, collagen-containing extracellular matrix, and collagen binding (**Figure 3C**). The findings demonstrate that both the ratio and the identities of FB1 have been altered in HLF compared with NLF.

Next, we explored the number of DEGs among the FB1 subpopulation and other fibroblast subpopulations in HLF. The heatmap demonstrated that the FB1 subpopulation exhibited a greater presence of genes like COL1A1, which are associated with collagen production, and transcription factors such as SCX, which are involved in the development of connective tissue, cell differentiation, and tissue repair (**Figure 3D**). KEGG analyses suggested that the DEGs in the FB1 subpopulation were associated with pathways such as the TGF-β signaling pathway, focal adhesion, ECM-receptor interactions, and the PI3K-Akt signaling pathway (**Figure 3E**), which have been proven to be crucial for the development of fibrotic lesions in the heart, lungs, and liver (25–30).

**Figure 4:**
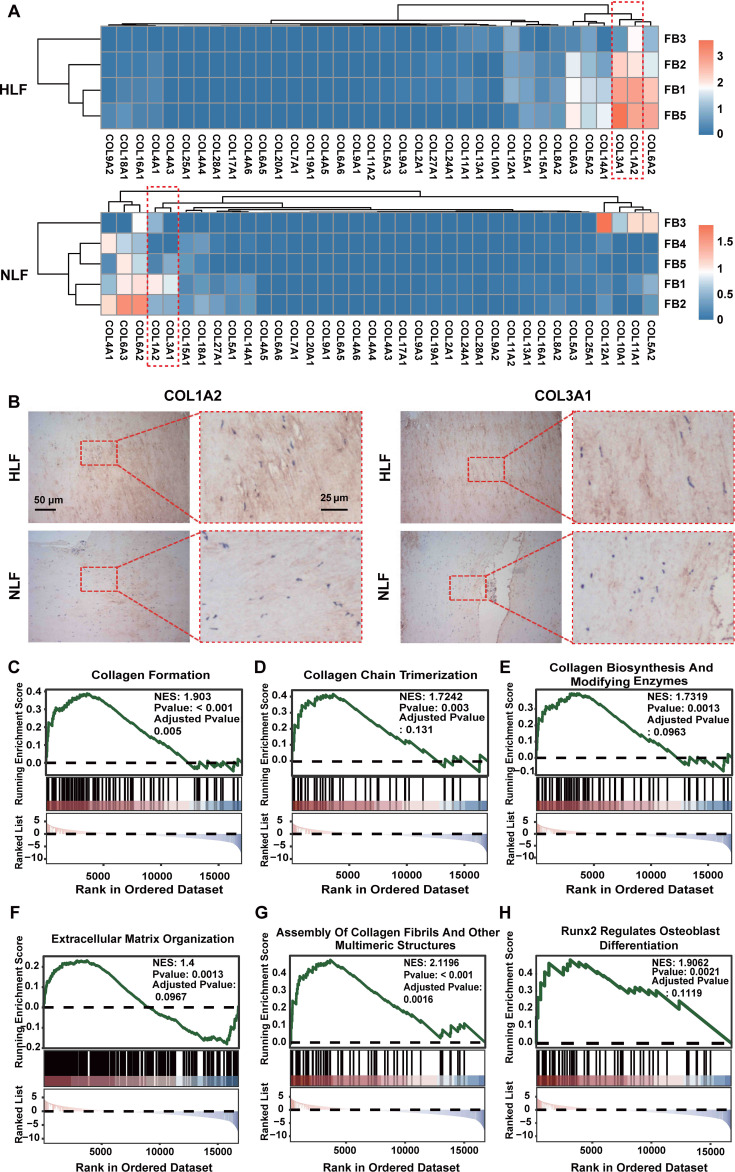
Collagen expression and GSEA enrichment analysis. **(A)** Collagen gene expression in HLF and NLF fibroblast subpopulations illustrated through a heatmap. The color red represents elevated gene expression, and blue represents reduced gene expression. **(B)** Images showing IHC staining of COL1A2 and COL3A1 in NLF and HLF specimens. Scale bar, 100 μm. **(C-H)** GSEA enrichment plots illustrating the activation of collagen-associated signaling pathways in HLF FB1 relative to NLF FB1 (P-value were showed in plots). IHC, immunohistochemistry; NLF, normal ligamentum flavum; HLF, hypertrophy of ligamentum flavum.

Moreover, DEG analysis of fibroblasts demonstrated that the expression levels of collagen-associated genes (COL1A2, COL3A1, COL1A1, and COL6A2) were markedly elevated in the HLF FB1 subpopulation as compared to other HLF fibroblast subpopulations (**Figure 4A**). IHC staining of LF also revealed that the HLF exhibited a higher quantity of positive cells and more extensive positive areas of COL1A2 and COL3A1 in comparison to the NLF (**Figure 4B**). GSEA analysis suggested that the FB1 subpopulation was highly associated with collagen formation (**Figure 4C**), collagen chain trimerization (**Figure 4D**), collagen biosynthesis and modifying enzymes (**Figure 4E**), extracellular matrix organization (**Figure 4F**), assembly of collagen fibrils and other multimeric structures (**Figure 4G**), and Runx2 regulates osteoblast differentiation (**Figure 4H**), and so on. These findings demonstrate that LF fibrosis is markedly related to the activation of the FB1 subpopulation.

### 3.4 Pseudotemporal ordering and RNA velocity analyses reveal the developmental trajectory of fibroblasts

**Figure 5:**
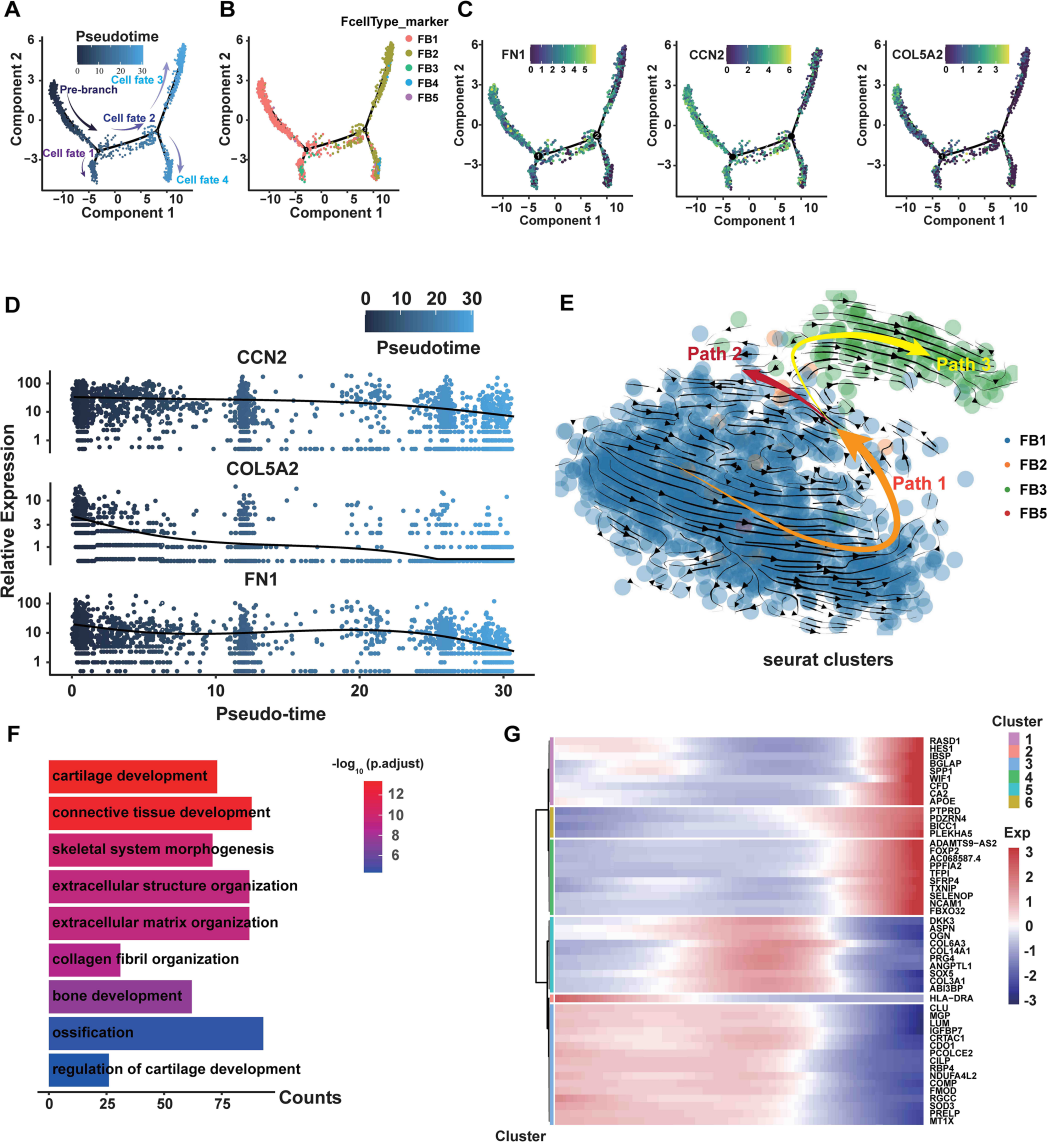
The transition from FB1 to FB3 is shown in the branching trajectory. **(A)** A branched trajectory is uncovered by ordering fibroblasts pseudo-temporally. **(B)** The five subpopulations are distributed on each branch. **(C)** The expression levels of FN1, CCN2, and COL5A2 in all fibroblasts are depicted in a diffusion map. **(D)** Scatterplot illustrating the expression changes of FN1, CCN2, and COL5A2 with pseudotiming. Pseudotimescales are shown on the horizontal coordinates, and gene expression levels are displayed on the vertical coordinates. **(E)** Through RNA velocity analysis, three velocity vector sets, such as Path1, Path2, and Path3, were differentiated along the diffusion-pseudotime. **(F)** GO bioprocess enrichment analysis focused on elevated genes in HLF FB1, comparing them with other fibroblast subpopulations in HLF (adjusted P-value of <0.05). **(G)** Gene expression changes with pseudotiming visualized on a heatmap. Clustering the differential genes on the heatmap allows for the identification of various sets of functional genes. NLF, normal ligamentum flavum; HLF, hypertrophy of ligamentum flavum.

To conduct a more detailed analysis of the associations among fibroblast subpopulations, we performed pseudotemporal ordering on all fibroblasts. The analysis identified a branched trajectory consisting of four primary branches: cell fate 1, cell fate 2, cell fate 3, and cell fate 4 (**Figure 5A**). Notably, the FB3 subpopulation was predominantly found in cell fate 1, and FB4 was chiefly located in the branches of cell fate 4. The majority of the cell fate 3 and 4 branches were made up of the FB2. FB1 and FB2 comprised the majority of the cell fate 2 branch (**Figure 5B**). FB1 primarily constituted the pre-branch and cell fate 2 branch, indicating the initial phases of fibroblasts (**Figure 5B**). Moreover, myofibroblast marker genes (FN1, CCN2, and COL5A2) are predominantly occupying the pre-branch portion (**Figure 5C**). The pseudotime analysis of gene expression indicated that FN1, CCN2, and COL5A2 were primarily expressed in the early and middle phases of cell development, with their expression levels declining in the later stages (**Figure 5D**).

Subsequently, we performed RNA velocity study on HLF fibroblasts to predict the potential direction as well as the rate of cellular state transitions. This study outlined three categories of vectors, referred to as paths. Additional analysis indicated that the paths displayed a branched trajectory with two main branches, Path2 and Path3, and a ‘pre-branch’ Path1, representing the initial phases of fibroblasts (**Figure 5E**). FB1 comprised the predominant portion of the pre-branch, while FB3 constituted the majority of Path3 (**Figure 5E**). Furthermore, GO analyses revealed that cartilage development, bone development, ossification, extracellular matrix organization and collagen fibril organization were more enriched in FB1 than in other fibroblast subpopulations (**Figure 5F**). Heatmaps of pseudo-temporal gene expression revealed that gene expression patterns changed at different stages of fibroblast differentiation (**Figure 5G**). In summary, these results demonstrate that FB1 might show lower differentiation compared to other fibroblast subpopulations, and the trajectory of fibroblasts displays a significant shift from the FB1 to the FB3 phenotype.

### 3.5 Changes in ligand-receptor interactions and signaling networks in HLF

**Figure 6:**
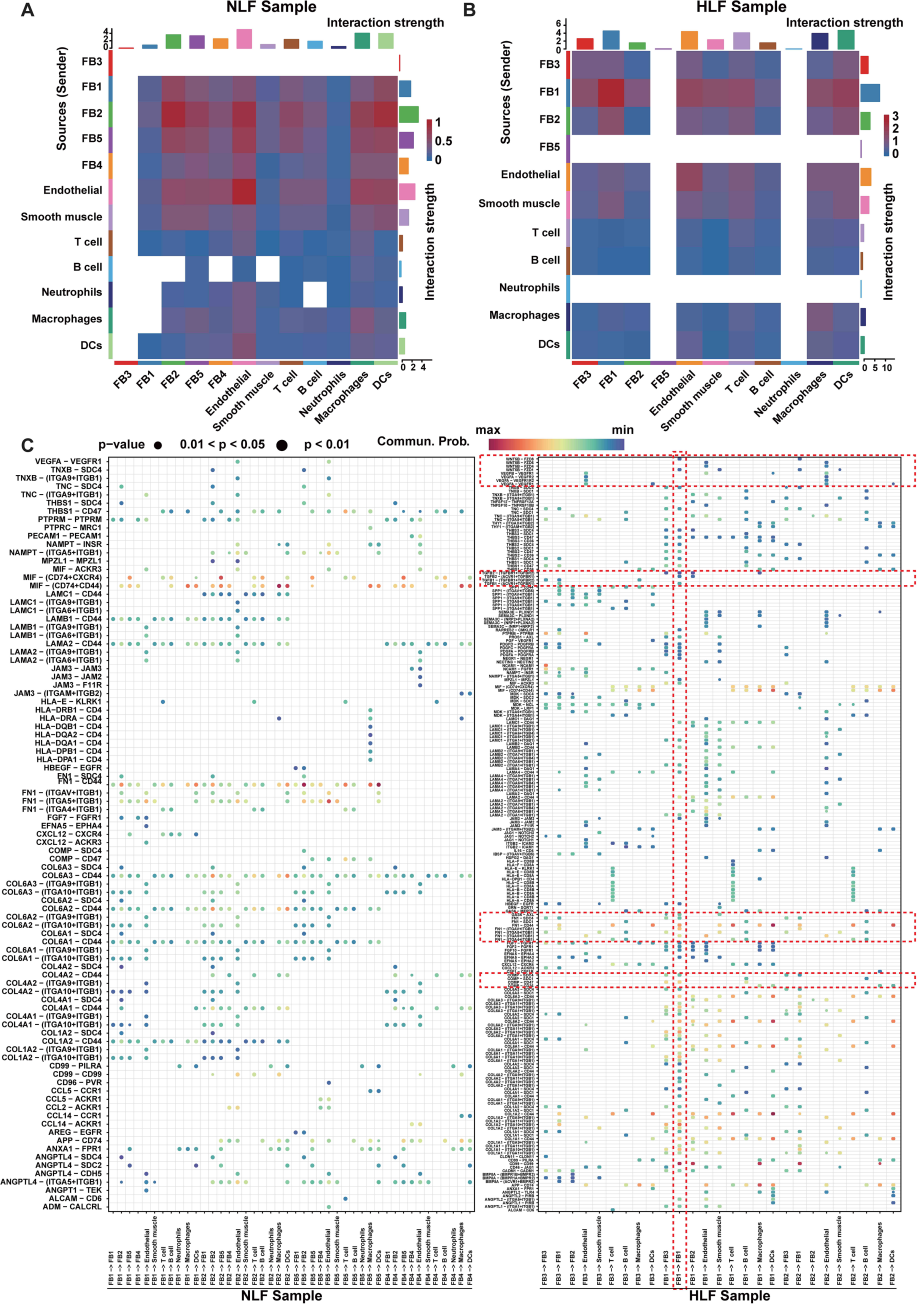
Analysis of potential interactions between ligands and receptors in FB1 fibroblasts. **(A, B)** Heatmaps displaying the intensity of intercellular interactions in NLF **(A)** and HLF **(B)**. **(C)** Significant specificity alterations in ligand-receptor pairs are observed between any fibroblast type and one of the cell lineages in both NLF and HLF. Dot plots reveal that receptors are expressed by all cell lineages, which also receive ligand signals from fibroblasts. NLF, normal ligamentum flavum; HLF, hypertrophy of ligamentum flavum.

To explore the communication network among fibroblast subpopulations and other cells in HLF and NLF, we conducted an analysis using CellPhoneDB 2.0 (31). We observed a dense communication network among fibroblasts and other cells in both HLF and NLF (**Figures 6A, B**). Fibroblasts were the primary drivers of intercellular communication in LF, as evidenced by the most prevalent and vigorous interactions among all four fibroblast subpopulations in both NLF and HLF. Briefly, FB2 interacted most frequently with other cells in NLF (**Figure 6A**). However, in HLF, FB1 had the most abundant interactions with themselves and other cells. In addition, the intensity of intercellular communication among FB1 cells was markedly elevated in HLF compared to NLF, highlighting the importance of FB1 interaction signaling in LF (**Figure 6B**).

Subsequently, we discovered ligand–receptor pairs among fibroblasts and other cell types, with fibroblast subpopulations sending ligand signals affecting all cell lineages (**Figures 6C, D**). The overall amount of significant interactions between ligand and receptor across cellular lineages was much higher in HLF compared to NLF, implying enhanced intercellular communication in the fibrosis environment. Furthermore, we discovered ligand-receptor pairs with significant differences. Certain ligands linked to fibrotic diseases, including COMP, FN1, and GAS6 in HLF, were not only up-regulated but also played crucial roles in FB1 intercellular communication compared to NLF. In addition, we observed that some membrane receptors like CD44, SDC4, AXL, and CD47, which were expressed by FB1, played a crucial role in intercellular communication. Importantly, several ligand-receptor pairs (COMP-SDC4, FN1-CD44, and GAS6-AXL) were more prominent in HLF, showing a unique modification of cell-cell interactions in the fibrotic condition.

**Figure 7:**
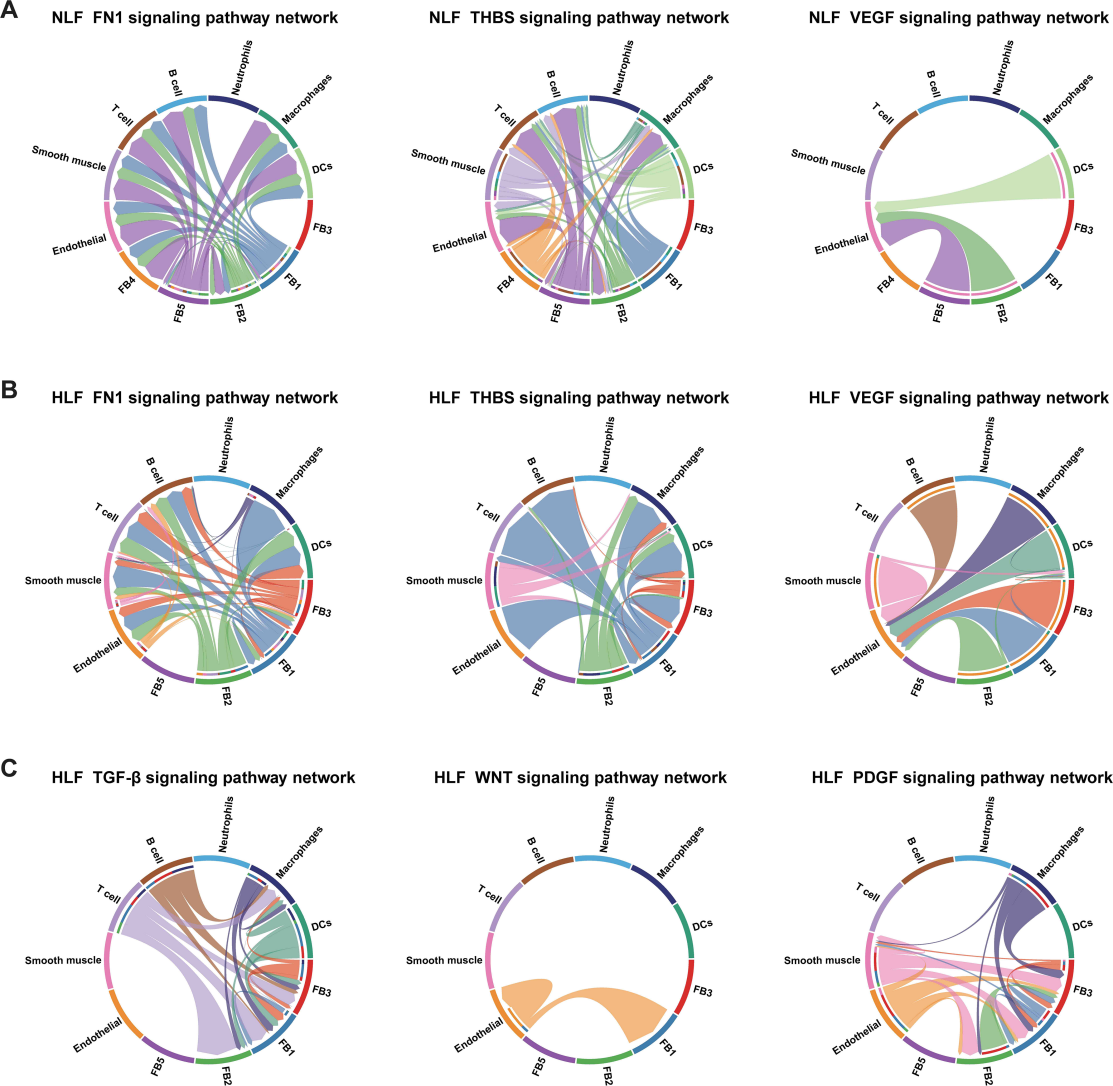
Principal networks of signaling pathways in LF. **(A, B)** Potential FN1, THBS, and VEGF signaling pathway networks involving fibroblasts and other cellular populations in NLF **(A)** and in HLF **(B)**. Every arrow is directed towards the receptors. A heatmap plot displays the average expression levels for each ligand and receptor. **(C)** The network of TGF-β, WNT, and PDGF signaling pathways interacts among fibroblasts and other cells in HLF. NLF, normal ligamentum flavum; HLF, hypertrophy of ligamentum flavum; DCs, dendritic cells.

Finally, we observed the changes in various signaling networks within LF. It was discovered that signaling networks such as FN1, THBS, VEGF, TGF-β, WNT, and PDGF, which were related to fibrotic diseases, showed significant upregulation (**Figures 7A, B**). Among them, the communication strength of FN1, THBS, and VEGF signaling networks was stronger in HLF than in NLF. In addition, the TGF-β, WNT, and PDGF signaling networks were exclusively found in HLF, indicating specific changes in the fibrotic state (**Figure 7C**).

### 3.6 LUM, CTSK, ASPN, OGN and MGP were significantly up-regulated in HLF FB1 and highly associated with the LF fibrosis

**Figure 8:**
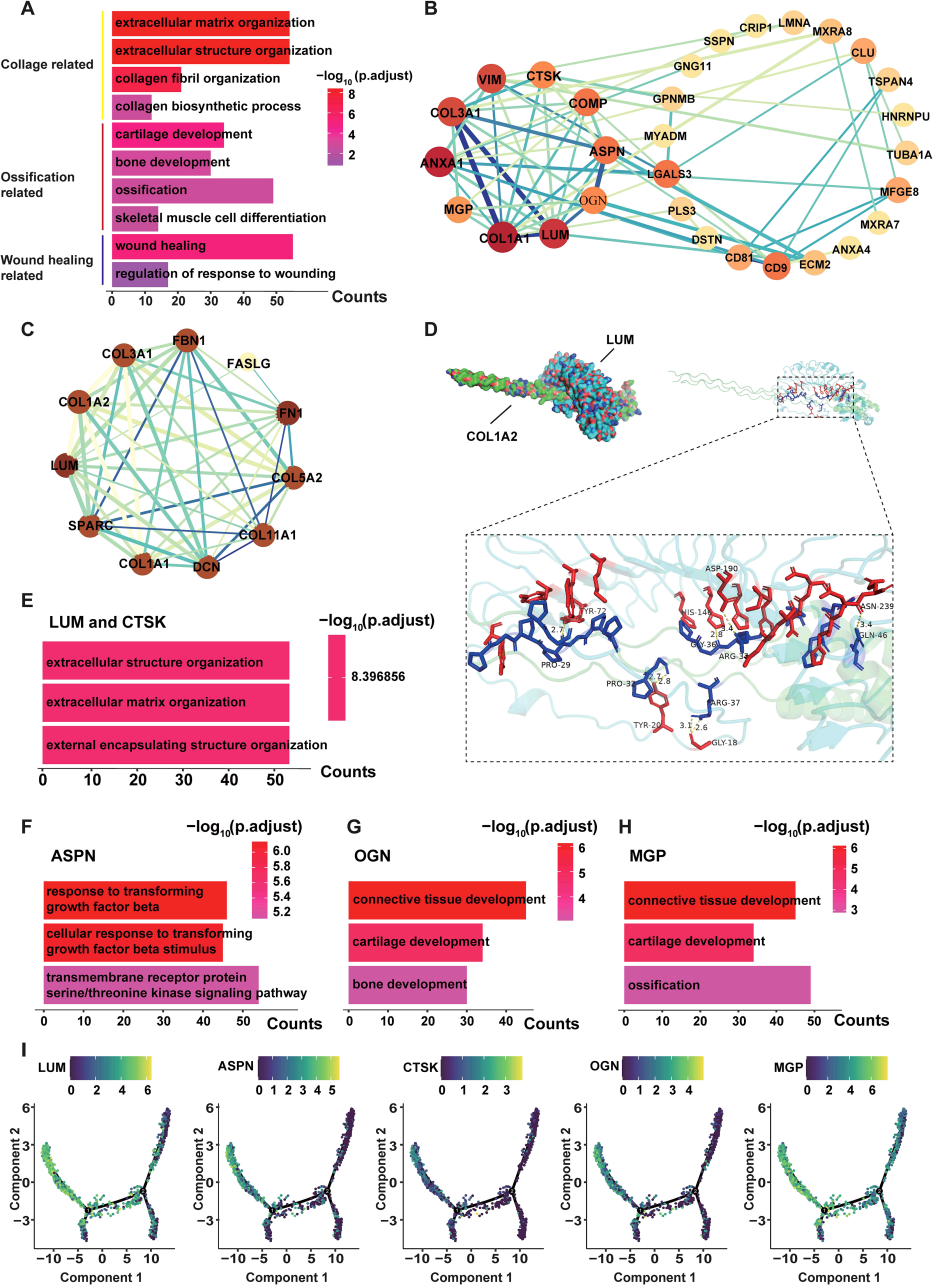
Hub genes in FB1 fibroblasts from HLF. **(A)** Genes that are upregulated in FB1 show functional enrichment, with an adjusted P-value under 0.05. **(B)** A PPI network featuring the 50 genes with the highest upregulation in HLF FB1. Fill the labels color with the “Degree” value, the redder the color, the higher the “Degree” value. The thicker and deeper the connecting lines between nodes, represent the stronger the gene interactions. Genes lacking protein interactions were excluded. **(C)** The PPI network of LUM. Displayed are proteins that interact with LUM. Fill the labels color with the “Degree” value, the redder the color, the higher the “Degree” value. The thicker and deeper the connecting lines between nodes, represent the stronger the gene interactions. **(D)** Using the HDOCK server, the binding mode of LUM to COL1A2 is predicted. LUM surrounds the side of COL1A2, and hydrogen bonds are shown as yellow dashes. **(E-H)** The genes LUM **(E)**, CTSK **(E)**, ASPN **(F)**, OGN **(G)**, and MGP **(H)** were among the top three contributors to the biological processes identified in the GO enrichment analysis. **(I)** The diffusion map presents the expression levels of LUM, CTSK, ASPN, OGN, and MGP in all fibroblasts. PPI, protein-protein interaction; NLF, normal ligamentum flavum; HLF, hypertrophy of ligamentum flavum.

GO enrichment analysis revealed that DEGs in HLF FB1 were mainly enriched in processes related to collagen, ossification, and wound healing (**Figure 8A**). Next, we concentrated on exploring the target genes in HLF FB1. The Cytoscape plug-in cytoHubba was utilized to analyze PPI networks for the identification of shared hub genes (32). The MCC algorithm identified 10 of the top 50 genes upregulated in HLF FB1 as potential hubs. After excluding individual genes, a PPI network of hub genes was built, comprising 30 nodes and 66 links, and Cytoscape was used to visualize it (**Figure 8B**, **Supplementary Table S3**). Excluding collagen-related genes, further analysis identified LUM, ASPN, COMP, CTSK, OGN, and MGP as the top-ranked hub genes, showing strong associations with tissue and organ fibrosis. Notably, LUM is the highest-ranked hub gene. Thus, we built and displayed the PPI network for LUM, highlighting a notable interaction with COL1A2 (**Figure 8C**). As is reported, by binding to collagen, LUM regulates the assembly and stabilization of collagen in organs and tissues, which contributes to the process of ECM in organs and tissues (33). In order to further predict the interacting mode between LUM and COL1A2, the hybrid docking strategy through the HDOCK server was used, and the results were shown in **Figure 8D**.

**Figure 9:**
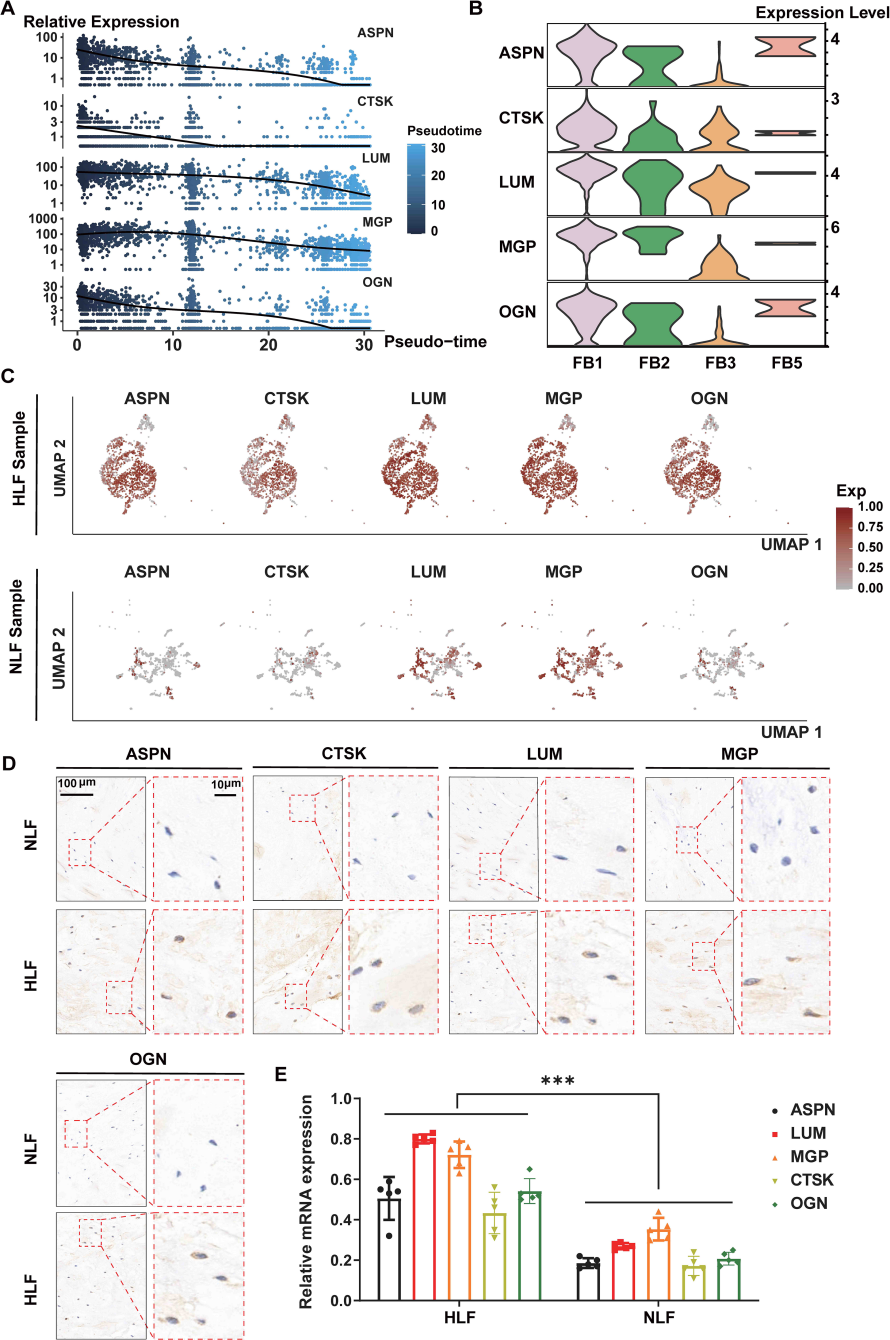
LUM, CTSK, ASPN, OGN, and MGP were significantly up-regulated in HLF. **(A)** Scatterplot depicting gene expression changes of LUM, CTSK, ASPN, OGN, and MGP with pseudotiming. The horizontal axis represents pseudotimescales, while the vertical axis indicates gene expression levels. **(B)** Violin plots displaying the expression levels of LUM, CTSK, ASPN, OGN, and MGP in HLF fibroblasts. **(C)** Expression feature plots for LUM, CTSK, ASPN, OGN, and MGP levels in both HLF and NLF fibroblasts. **(D)** IHC staining images showing the expression levels of LUM, CTSK, ASPN, OGN, and MGP in NLF and HLF specimens. **(E)** Detection of the mRNA-expression levels of LUM, CTSK, ASPN, OGN, and MGP in NLF and HLF specimens (n=5). ^***^P < 0.001. IHC, immunohistochemistry; NLF, normal ligamentum flavum; HLF, hypertrophy of ligamentum flavum.

Furthermore, GO was used to analyze biological processes enriched in the genes of LUM, CTSK (**Figure 8E**), ASPN (**Figure 8F**), OGN (**Figure 8G**), and MGP (**Figure 8H**). The main function of these genes was to directly or indirectly participate in the development of connective tissue. Through pseudotiming analysis, we found that these genes predominantly occupied the pre-branch (**Figure 8I**), and MGP, ASPN, OGN, and LUM were mainly expressed in the initial and middle stages of cell development and gradually declined in the late stages (**Figure 9A**), while CTSK was mainly expressed at the early stage (**Figure 9A**). Both the violin plots (**Figure 9B**) and the UMAP (**Figure 9C**) showed that MGP, ASPN, OGN, LUM, and CTSK were notably expressed in HLF FB1 compared to other fibroblast subpopulations in HLF. Histologically, IHC staining revealed a higher number of positive cells and a larger positive area for MGP, ASPN, OGN, LUM, and CTSK in HLF compared to NLF (**Figure 9D**), which was also confirmed by RT-PCR results (**Figure 9E**). Collectively, these findings indicated that HLF FB1 showed a marked up-regulation of LUM, CTSK, ASPN, OGN, and MGP, which were highly correlated with LF fibrosis.

### 3.7 Gene regulatory network comparison highlights the key regulatory transcription factors in HLF fibroblasts

**Figure 10:**
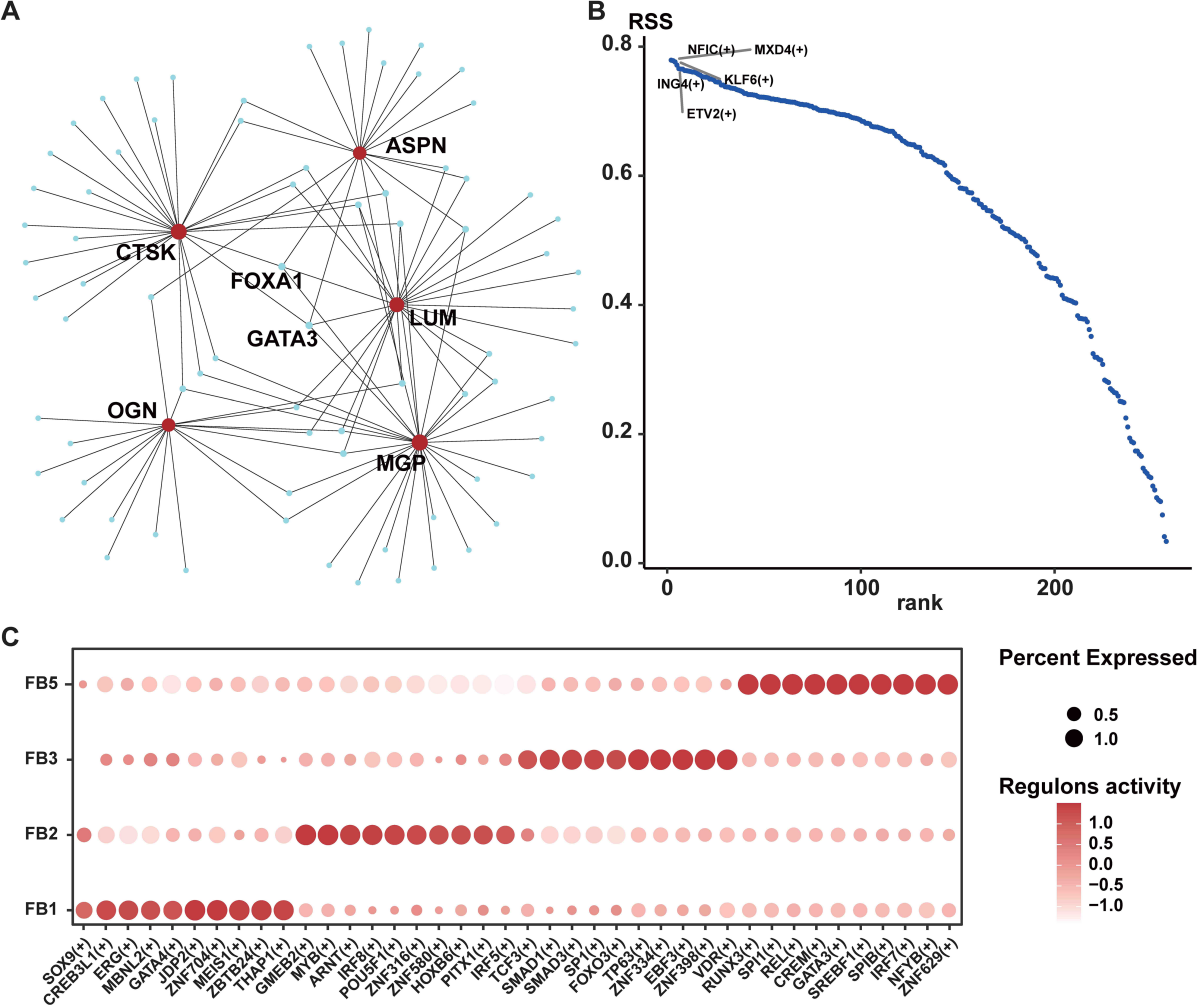
The regulatory network of transcription factors in HLF FB1. **(A)** Transcription factors and hub genes regulatory network. The small light blue circles symbolize transcription factors, while the red circles denote hub genes. **(B)** Transcript-specific regulon in HLF FB1. Higher RSS scores suggest a stronger link between the regulon and HLF FB1. **(C)** Dot plots displaying the expression of selected master transcription factors within each fibroblast subpopulation. The proportion of cells expressing the gene is indicated by the circle’s size, and the expression level is shown by the color intensity. FB1, mesenchymal fibroblasts. NLF, normal ligamentum flavum; HLF, hypertrophy of ligamentum flavum.

The functions and roles of cells are largely determined by transcription factors (TFs) (34). In order to explore the key TFs interacting with the five key genes of MGP, ASPN, LUM, and CTSK, we used NetworkAnalyst to predict the related TFs and visualized the TF-gene regulatory network through Cytoscape. As a result, we found that the five hub genes might have their expression regulated through interactions with the TFs of FOXA1 and GATA3 (**Figure 10A**). Furthermore, to shed light on the regulatory factors linked to the FB1 subpopulation, we carried out SCENIC analysis and identified five specific TFs in FB1, namely NFIC, MXD4, KLF6, ING4, and ETV2 (**Figure 10B**). Subsequently, we analyzed the variability of TFs in HLF. The heatmap illustrated the varying expression levels of TFs in fibroblasts derived from HLF. In brief, several skeletal system and fibrosis-associated TFs, such as GATA4, SOX9, ERG, MBNL2, and CREB3L1, exhibited high expression levels in FB1 (**Figure 10C**), aligning with its mesenchymal traits. Nevertheless, other TFs related to skeletal development, cartilage differentiation, and ECM formation and remodeling, including TCF3, SMAD1, SMAD3, SP1, and FOXO3, were abundant in the FB3 (**Figure 10C**). These analyses concluded that the expression pattern of TFs shifted as the fibroblast phenotype transitioned from FB1 to FB3, indicating that CREB3L1, SOX9, ERG, MBNL2, and GATA4 were crucial in the initial phases of LF fibrosis, while TCF3, SMAD1, SMAD3, SP1, and FOXO3 were more involved in the later stages. Among these TFs, SOX9 has been demonstrated to have a crucial regulatory function in fibrogenesis and organ fibrosis, including the liver, kidney, and heart (35–37). Therefore, further studies are necessary to clarify its role in LF fibrosis.

### 3.8 Cellular heterogeneity and regulatory changes in HLF AECs

**Figure 11:**
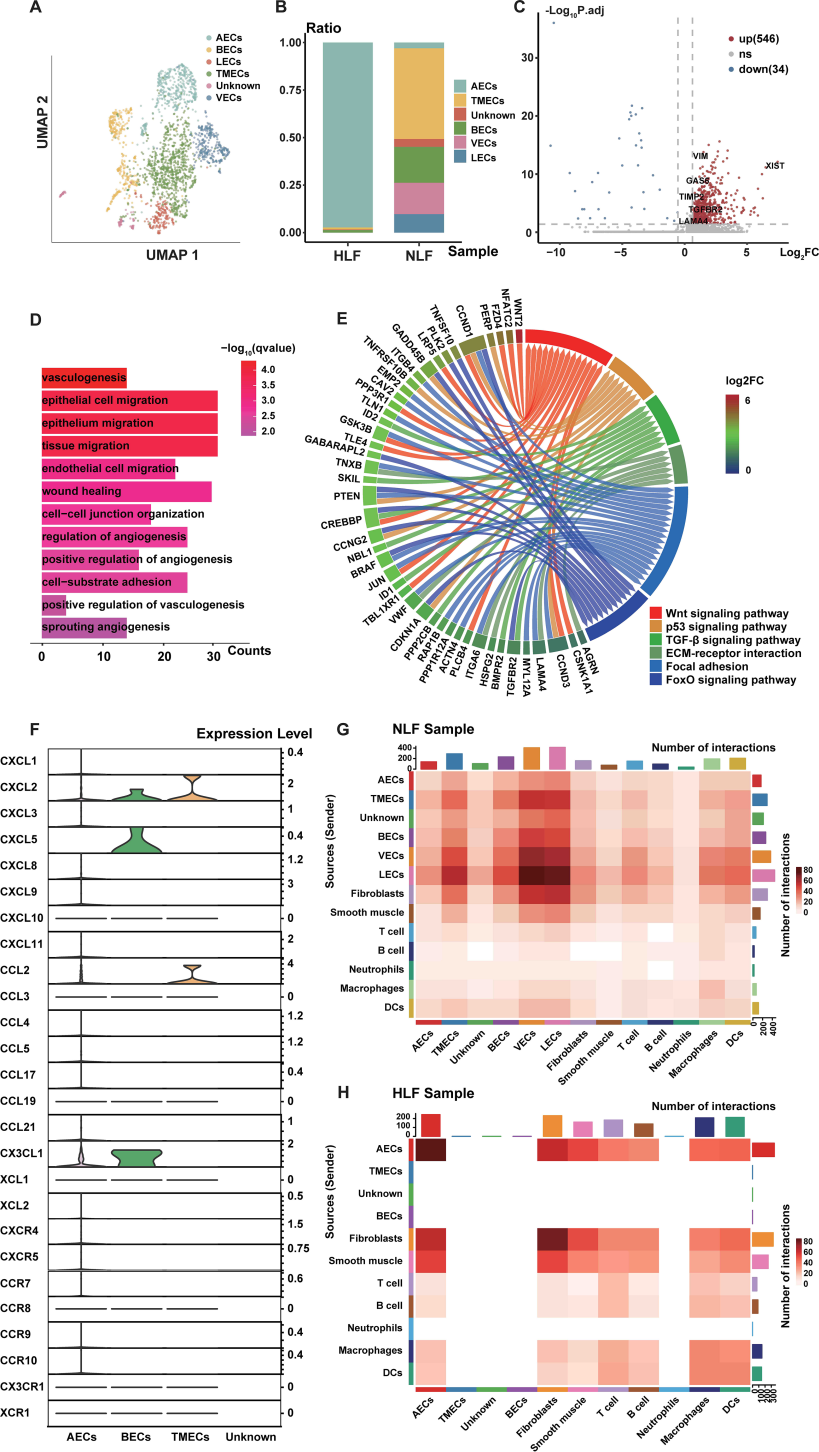
Characteristics of AECs in LF fibrosis. **(A)** Six distinct subpopulations of ECs were identified in LF specimens. **(B)** Comparison of the percentage of six EC subpopulations in NLF and HLF. **(C)** Volcano plot showing differentially expressed genes in arterial ECs, with 546 genes upregulated and 34 downregulated. **(D)** GO enrichment analysis on up-regulated genes among differentially expressed genes in AECs between NLF and HLF. P-value under 0.05 was set as the threshold for significance. **(E)** KEGG enrichment analysis of genes that are up-regulated in differentially expressed genes in AECs between NLF and HLF. **(F)** Plots in the form of violins illustrating the expression of genes linked to ‘chemotaxis activity’. **(G, H)** Heatmaps presenting the degree of intercellular communication in NLF **(D)** and HLF **(E)**. AECs, arterial endothelial cells; NLF, normal ligamentum flavum; HLF, hypertrophy of ligamentum flavum; EC, endothelial cell.

In order to better grasp the function of ECs in HLF, we further analyzed a total of 2,071 ECs, comprising 364 from HLF and 1,707 from NLF. Hierarchical cluster analysis showed ECs could be classified into six subpopulations: AECs, blood endothelial cells (BECs), lymphatic endothelial cells (LECs), venous endothelial cells (VECs), tumor microvascular endothelial cells (TMECs), and unknown (**Figure 11A**). The HLF failed to detect the LECs and VECs subpopulations, and the unknown ECs subpopulation contained very few cells. Therefore, we excluded it from the following analyses. To begin with, we assessed the percentages of the six EC subpopulations between HLF and NLF. The findings indicated that the percentage of AECs subpopulation was significantly higher in HLF than in NLF (**Figure 1B**). In addition, the analysis of DEGs from ECs indicated that the AECs subpopulation had the highest number of DEGs (total: 580 genes; up: 546; down: 34), suggesting that this subpopulation experiences substantial alterations during fibrosis (**Figure 11C**).

Therefore, our further exploration concentrated on AECs. It was found that several fibrosis-related genes, such as XIST, GAS6, TGFBR2, and TIMP2, exhibited significantly higher expression levels in HLF AECs than in NLF AECs. (**Figure 11C**). Next, we performed GO and KEGG analyses in AECs (**Figures 11D, E**). These findings revealed that some biological processes, such as vasculogenesis, cell migration, cell-substrate adhesion, regulation of angiogenesis, wound healing, and cell-cell junction organization (**Figure 11D**), as well as the WNT, FoxO, and TGF-β signaling pathways (**Figure 11E**), were enriched in the HLF AECs subpopulation. In addition, the HLF AECs subpopulation exhibits elevated expression of ECM-related genes, including TGFBR2, LAMA4, and IGFBP7, indicating potential links between the AECs subpopulation and ECM-producing cells (**Figure 11C**).

Chemokines are essential in fibrotic diseases by engaging in cellular communication and inflammatory responses (38). Thus, to further explore the heterogeneity of EC subpopulations, we identified chemotactic activity-related gene expression levels. Violin plots showed that BECs had significant expression of CXCL2, CXCL5, and CX3CL1, whereas CXCL2 and CCL2 were highly expressed in TMECs (**Figure 11C**). Interestingly, chemokine gene expression levels were low in AECs. In addition, emerging evidence indicates that endothelial-to-mesenchymal transition (EndMT) contributes to fibrosis in various organs (21, 22, 39, 40). Similarly, analysis of EndMT-related gene expression levels revealed that the HLF AECs subpopulation had considerably higher VIM expression than the NLF AECs subpopulation (**Figure 11C**).

**Figure 12:**
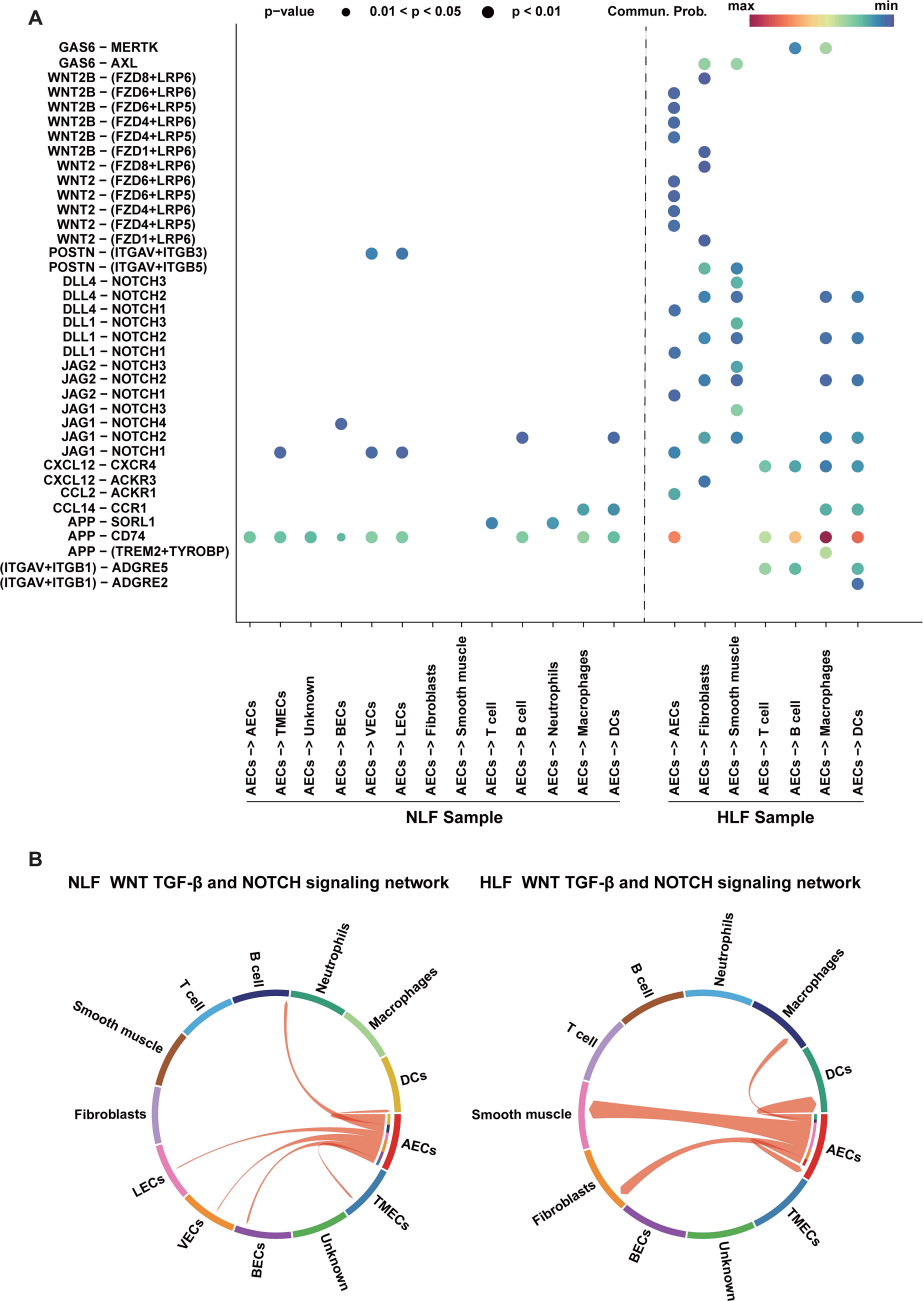
Analysis of potential interactions between ligands and receptors in AECs. **(A)** Significant changes in ligand-receptor pairs among ECs and all cell lineages were observed in NLF and HLF. Dot plots indicate that every cell lineage expresses receptors and receives ligand signals from ECs. **(B, C)** Potential WNT, TGF-β, and NOTCH signaling pathway network among ECs and all cell lineages in NLF **(B)** and HLF **(C)**, with all arrows directed towards the receptors. ECs, endothelial cells; NLF, normal ligamentum flavum; HLF, hypertrophy of ligamentum flavum; AECs, arterial endothelial cells; BECs, blood endothelial cells; VECs, venous endothelial cells; TMECs, tumor microvascular endothelial cells; LECs, lymphatic endothelial cells; DCs, dendritic cells.

Furthermore, to gain a deeper insight into ECs in HLF, we conducted a CellPhoneDB 2.0 analysis (31), revealing ligand-receptor interactions between ECs and other cell types. The interaction network analysis indicated possible communication among DCs, smooth muscle, fibroblasts, macrophages, and ECs (**Figures 11G, H**). In NLF, communication between VECs and LECs was most significant (**Figure 11G**). Notably, it was found that AECs and fibroblasts were dominating the intercellular communication in HLF (**Figure 11H**). In addition, some ligand-receptor pairs like GAS6-AXL, WNT-(FZD+LRP), DLL-NOTCH, JAG-NOTCH, CXCL12-CXCR4, and APP-CD74 showed stronger interactions in HLF, indicating the enhanced intensity of intercellular communication in fibrotic conditions (**Figure 12A**). Further analysis showed that the WNT, TGF-β, and NOTCH signaling networks showed meaningful enhancement in HLF compared to NLF (**Figure 12B**). Importantly, some EndMT pathways, including the WNT and NOTCH pathways, were also significantly modified (**Figure 12C**). In summary, our work identified HLF EC heterogeneity for the first time, and the results indicated that ECs might be involved in LF fibrosis by altering chemokine expression, promoting EndMT and angiogenesis.

## 4 Discussion

LSS is a prevalent spinal surgical disease that predominantly impacts the ligaments and discs related to the spine, potentially resulting in chronic low back pain, claudication, and various clinical manifestations. It exhibits a significant frequency among the elderly population (1, 2, 4). LF fibrosis is the main pathological feature of LSS (4). Previous studies have revealed that there are multiple factors that have been considered in association with LF fibrosis, including aging, excessive mechanical stress, inflammatory response and trauma (7, 13). Mechanistically, the TGF-β/Smad, Wnt/β-catenin, NF-κB, and Hedgehog pathways have been identified as significant contributors to LF fibrosis (7, 8, 10, 13). However, the main target genes and precise regulatory mechanisms driving its progression of LF fibrosis are still not well understood. Here, we established a representative lumbar LF single-cell atlas and analyzed the heterogeneity as well as primarily regulatory pathways of fibroblasts and ECs, which enabled us to identify the pathological mechanisms driving LF fibrosis. This information will enhance our comprehension of LF fibrosis etiology.

Fibroblasts are widely acknowledged as a crucial cell type in numerous fibrotic diseases (23, 24, 41). Here, we identified five fibroblast subpopulations in human LF utilizing the scRNA-seq technique (**Figure 2B**). In HLF, the proportion of FB1 was significantly higher (**Figure 2E**). According to our results, genes exhibiting heightened expression in HFL FB1 are linked to bone and cartilage development, ossification, and chondrocyte differentiation, indicating FB1 has a stronger mesenchymal element (**Figure 3C**). FB1 also exhibited higher levels of several master transcription factors (TFs) associated with skeletal system development, such as CREB3L1 and SOX9 (42, 43) (**Figure 10C**). The high expression of these TFs and genes in HLF FB1 determines specific cellular characteristics of FB1, indicating the multiple biological functions of the FB1 subpopulation in LF fibrosis.

Further analysis identified some fibrosis-related genes, such as MGP (44), ASPN (45, 46), OGN (47, 48), LUM (49, 50), and CTSK (51–54), that exhibited high expression in HLF compared to NLF (**Figure 3B**), showing that they might play a critical role in LF fibrosis. These genes have been identified as significant causative factors in the progression of fibrotic diseases in other tissues or organs, according to related studies (29, 55, 56). Nonetheless, these studies did not investigate the cellular origin of these proteins. Here, both the violin plots (**Figure 9B**) and the UMAP (**Figure 9C**) showed that MGP, ASPN, OGN, LUM, and CTSK were notably expressed in HLF FB1 compared to other fibroblast subpopulations in HLF. Histologically, IHC staining also revealed a higher number of positive cells and a larger positive area for these proteins in HLF (**Figure 9D**). Overall, our results demonstrated that HLF FB1 exhibited a significant increase in certain secretory proteins strongly linked to HLF, highlighting the important role of the FB1 subpopulation in LF fibrosis. Currently, surgery is the primary treatment for LSS (4). Based on the above results, we could create strategies such as using small molecule inhibitors of MGP, ASPN, OGN, LUM, and CTSK to target FB1. Drugs aimed at specific FB1 targets for LSS treatment show considerable promise.

Analysis of intercellular communication demonstrated that all cell lineages and fibroblasts had a dense communication network (**Figures 6A, B**). Notably, in HLF, FB1 interacted most abundantly with other cells, indicating its predominance in intercellular communication in the context of LF fibrosis (**Figure 6B**). This once again confirmed the importance of FB1 in LF fibrosis. As is well demonstrated, TGF-β promotes fibroblast activation, extracellular matrix (ECM) synthesis, and the transformation of fibroblasts into myofibroblasts, crucial for fibrogenesis (57). Also, we found a marked increase of TGF β-TGFR receptor interactions in HLF FB1 compared to NLF (**Figure 6C**), indicating the central roles of the TGF-β pathway in LF fibrosis. Additionally, we discovered that LF fibrosis was linked with an increase in a few previously documented fibrosis-related interactions, such as WNT and THBS ligand–receptor interactions (58–60) (**Figure 6C**). Interestingly, there was a marked increase in ligand-receptor pairs for some interactions involving FB1 and other cell lineages in HLF, such as COMP-SDC4, FN1-CD44, and GAS6-AXL. Thus, COMP, FN1, and GAS6 could be considered promising therapeutic targets.

As is well known, myofibroblasts greatly increase in number and cause collagen production in fibrotic diseases. A dynamic fibroblast-myofibroblast transition occurs in response to tissue injury (24, 30). Transient activation of myofibroblasts contributes to tissue repair, whereas persistent activation triggers pathological fibrosis (24, 30). In fibrosis, the transition from fibroblasts to myofibroblasts, which generate ECM and release factors related to fibrosis, is a crucial event that drives the fibrotic response (24, 30, 61, 62). Here, we observed that myofibroblast markers (FN1, CCN2, and S100A4) were markedly upregulate in the HLF FB1 subpopulation as compared to NLF FB1 (**Figure 3B**). To explore the correlation between myofibroblasts and FB1 in LF fibrosis, we performed a pseudotime analysis. The analysis revealed that myofibroblast marker genes (FN1, CCN2, and COL5A2) were highly expressed mainly in the pre-branch portion where FB1 is located (**Figure 5C**). In addition, pseudotime analysis of gene expression also showed that FN1, CCN2 and COL5A2 were also expressed predominantly at early stages of cell development (**Figure 5D**). These results reveal that myofibroblasts play an important role in the early stages of possible ligamentum flavum fibrosis progression. Further analysis of the expression level of the myofibroblast marker FN1 in fibroblasts revealed that the proportion of myofibroblasts was higher in HLF than in NLF (95.34% *vs*. 60%) (**Supplementary Figure 1A**), which revealed that the proportion of myofibroblasts increased during the progression of LF fibrosis. Furthermore, in HLF, myofibroblasts were enriched in the FB1 subpopulation (86.50%) and existed in the other three subpopulations (8.82%) (**Supplementary Figure 1A**). These findings revealed that a portion of FB1 were myofibroblasts; the majority of myofibroblasts were in the FB1 subpopulation in HLF. In summary, we conclude that FB1 may be transformed into myofibroblasts during LF fibrosis, thereby promoting the progression of LF fibrosis. Similar conclusions were obtained in the study of fibrotic skin disease conducted by Deng et al. They results suggested that part of mesenchymal fibroblasts were myofibroblasts, and most of the myofibroblasts were in the mesenchymal fibroblast subpopulation in keloid (18).

Recently, growing evidence suggests that ECs play multiple key roles in the progression of fibrotic diseases (63, 64). ECs drive fibrosis through multidimensional mechanisms such as EndMT, pro-fibrotic factor release, inflammatory regulation, vascular dysfunction and intercellular communication (64–66). Therefore, we also explored the specific regulatory alterations of ECs occur in LF fibrosis. Our data demonstrated the high degree of heterogeneity of ECs in HLF. Furthermore, we found that ECs might be involved in LF fibrosis by altering chemokine expression, promoting EndMT and angiogenesis, and AECs played a crucial role in LF fibrosis.

There are several limitations to this study. Firstly, due to various reasons, each group only has one specimen, indicating that larger specimens are needed to validate these findings. Secondly, the progression of fibrosis is influenced by various cells. Here, we only analyzed two main cells of fibroblasts and endothelial cells; whether other cells are involved in HLF is an area of investigation that needs to be further explored.

## 5 Conclusion

Our systematic analysis establishes an LF single-cell atlas in LSS and LDH. Moreover, we conduct a systematic investigation of the EC and fibroblast heterogeneity in HLF at the single-cell level and identify a substantial increase in the FB1 and AECs subpopulation in HLF, which are crucial for the progression of LF fibrosis. These findings will enhance the understanding of HLF pathogenesis and identify potential targets for the treatment of LSS.

## Data Availability

The data presented in the study are deposited in the GEO repository, accession number GSE294458.
