# Supplementary material for: Single-cell RNA-seq uncovers lineage-specific regulatory alterations of fibroblasts and endothelial cells in ligamentum flavum hypertrophy
Source: Front Immunol. 2025 May 15;16:1569296. doi: 10.3389/fimmu.2025.1569296 (PMC12119296; doi:10.3389/fimmu.2025.1569296)
Supplement: Supplementary file 1 [file DataSheet1.docx]

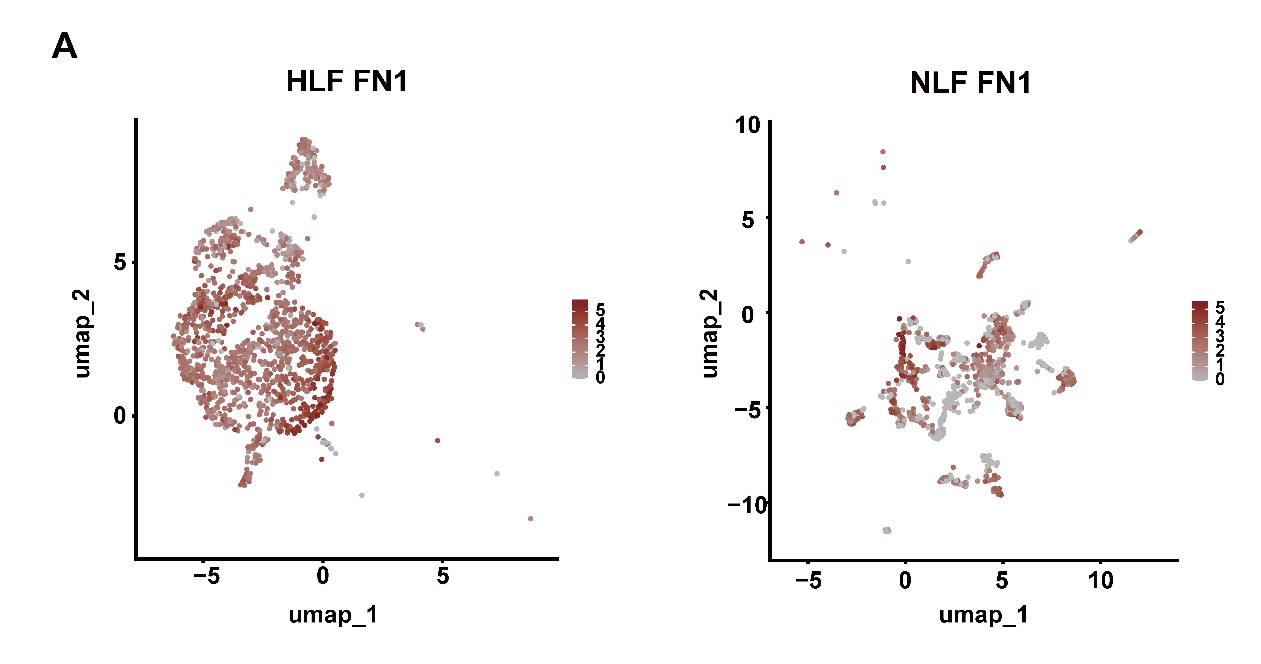
**Supplementary Figure 1. Most of myofibroblasts were in the FB1 subpopulation. (A)** Feature plots of expression distribution for FN1 in HLF and NLF. Expression levels for each cell are color-coded and overlaid onto the UMAP plot.
