# Supplementary material for: Single-cell RNA-seq uncovers lineage-specific regulatory alterations of fibroblasts and endothelial cells in ligamentum flavum hypertrophy
Source: Front Immunol. 2025 May 15;16:1569296. doi: 10.3389/fimmu.2025.1569296 (PMC12119296; doi:10.3389/fimmu.2025.1569296)
Supplement: Supplementary file 2 [file Table1.docx]

Supplementary Material

**Single-Cell RNA-Seq uncovers lineage-specific regulatory alterations of fibroblasts and endothelial cells in ligamentum flavum hypertrophy**

**Table S1** Information of the clinical sample for single-cell RNA sequencing

|  | Patient 1 | Patient 2 |
| --- | --- | --- |
| Age (years) | 58 | 60 |
| Gender | Female | Female |
| BMI | 23.4 | 22.8 |
| Smoking | NO | NO |
| Diagnosis | LDH | LSCS |
| LF thickness (mm) | 2.87 | 5.89 |
| Comorbidities/medical history | NO | NO |
| Sample Type | NFL | HLF |
| Sample Level | L4-L5 | L4-L5 |

**[Table](" \l "tbl1#tbl1) S2** RT-PCR primers used in this study

| Primer | Sequence |
| --- | --- |
| MGP | Forward: 5′ -GGACTAGGGCACAGGTTTGA-3′  Reverse: 5′ -TGGGTTTGGTCCACAGACAG-3′ |
| ASPN | Forward: 5′ -TGCGAAGGCTGTATCTGTCC-3′  Reverse: 5′ -GAACACCGTCACCCCTTCAA-3′ |
| OGN | Forward: 5′ -AGCTACAACGGGAAAAAGAGAGT-3′  Reverse: 5′ -GAGGCACAAGCAGTAACAGG-3′ |
| LUM | Forward: 5′ -TCCTGGCATTGATTGGTGGT-3′  Reverse: 5′ -CTTGGGTAGCTTTCAGGGCA-3′ |
| CTSK | Forward: 5′ -CCCGCAGTAATGACACCCTT-3′  Reverse: 5′ -AAAGCCCAACAGGAACCACA-3′ |
| GAPDH | Forward: 5′ -AGAAGGTGGTGAAGCAGGCGTC-3′  Reverse: 5′ -AAAGGTGGAGGAGTGGGTGTCG-3′ |

**Table S3** The top 10 hub genes in FB1 ranked by MCC method

| Rank | Name | Score |
| --- | --- | --- |
| 1 | COL1A1 | 215 |
| 2 | LUM | 210 |
| 3 | COL3A1 | 200 |
| 4 | ASPN | 150 |
| 5 | COMP | 146 |
| 6 | CTSK | 121 |
| 7 | OGN | 54 |
| 8 | MGP | 48 |
| 9 | ANXA1 | 31 |
| 10 | VIM | 27 |
